# Diversity and evolution of the *Wolbachia* endosymbionts of *Bemisia* (Hemiptera: Aleyrodidae) whiteflies

**DOI:** 10.1002/ece3.1126

**Published:** 2014-06-11

**Authors:** Xiao-Li Bing, Wen-Qiang Xia, Jia-Dong Gui, Gen-Hong Yan, Xiao-Wei Wang, Shu-Sheng Liu

**Affiliations:** Ministry of Agriculture Key Laboratory of Agricultural Entomology, Institute of Insect Sciences, Zhejiang UniversityHangzhou, 310058, China

**Keywords:** *Bemisia tabaci*, FISH, horizontal transmission, multilocus sequence typing, vertical transmission, whiteflies, *Wolbachia*

## Abstract

*Wolbachia* is the most prevalent symbiont described in arthropods to date. *Wolbachia* can manipulate host reproduction, provide nutrition to insect hosts and protect insect hosts from pathogenic viruses. So far, 13 supergroups of *Wolbachia* have been identified. The whitefly *Bemisia tabaci* is a complex containing more than 28 morphologically indistinguishable cryptic species. Some cryptic species of this complex are invasive. In this study, we report a comprehensive survey of *Wolbachia* in *B. tabaci* and its relative *B. afer* from 1658 insects representing 54 populations across 13 provinces of China and one state of Australia. Based on the results of PCR or sequencing of the 16S rRNA gene, the overall rates of *Wolbachia* infection were 79.6% and 0.96% in the indigenous and invasive *Bemisia* whiteflies, respectively. We detected a new *Wolbachia* supergroup by sequencing five molecular marker genes including 16S rRNA, *groEL*, *gltA*, *hcpA*, and *fbpA* genes. Data showed that many protein-coding genes have limitations in detecting and classifying newly identified *Wolbachia* supergroups and thus raise a challenge to the known *Wolbachia* MLST standard analysis system. Besides, the other *Wolbachia* strains detected from whiteflies were clustered into supergroup B. Phylogenetic trees of whitefly mitochondrial cytochrome oxidase subunit I and *Wolbachia* multiple sequencing typing genes were not congruent. In addition, *Wolbachia* was also detected outside the special bacteriocytes in two cryptic species by fluorescence *in situ* hybridization, indicating the horizontal transmission of *Wolbachia*. Our results indicate that members of *Wolbachia* are far from well explored.

## Introduction

*Wolbachia* are rickettsial endosymbiotic bacteria in the class Alphaproteobacteria. *Wolbachia* bacteria are considered the most widespread endosymbionts in animals as they are found in all major classes of arthropods and some nematodes (Jeyaprakash and Hoy 2000; Werren and Windsor 2000; Duron et al. 2008; Russell et al. 2012). A meta-analysis suggests that the proportion of *Wolbachia* infection in insect species in the terrestrial world is about 40% (Zug and Hammerstein 2012).

In some host species, the successful maintenance and spread of *Wolbachia* is mainly achieved by the induction of cytoplasmic incompatibility to produce more female offspring, thus enhancing its maternal transmission (Stouthamer et al. 1999). In addition, manipulation of reproduction by *Wolbachia* includes feminizing genetic males, causing parthenogenesis, and killing male progenies (Stouthamer et al. 1999; Werren et al. 2008). Recent studies found that *Wolbachia* benefits insect hosts by providing essential nutrition (Hosokawa et al. 2010), enhancing host stem cell's proliferation (Fast et al. 2011), and protecting insect from pathogenic RNA viruses (Hedges et al. 2008).

The genus *Wolbachia* is highly divergent and has so far been divided into 13 supergroups (A-N, except for G which is a combination of A and B) (Lo et al. 2002, 2007; Baldo and Werren 2007; Haegeman et al. 2009; Ros et al. 2009; Augustinos et al. 2011). *Wolbachia* supergroups are characterized mainly with molecular markers such as *rrs* (16S rRNA), *ftsZ* (cell division protein), *gltA* (Citrate synthase), *groEL* (Chaperonin GroEL) and *wsp* (*Wolbachia* surface protein) genes (O'Neill et al. 1992; Zhou et al. 1998; Werren and Windsor 2000; Casiraghi et al. 2005). *Wolbachia* genotyping is inferred mainly from multi locus sequence typing (MLST) genes (*gatB*, *coxA*, *hcpA*, *fbpA*, and *ftsZ* genes) and amino acid sequences of the four hypervariable regions (HVRs) of WSP protein (Baldo et al. 2005, 2006).

*Bemisia tabaci* (Hemiptera: Aleyrodidae) is a complex containing more than 28 morphologically indistinguishable cryptic species (De Barro et al. 2011; Hu et al. 2011). Through millions of years of evolution, the various cryptic species of this complex show a clear geographic pattern of distribution around the globe (Boykin et al. 2007, 2013; De Barro et al. 2011). However, with the development of modern transport, whiteflies have been transferred frequently among different continents (Naranjo et al. 2010). During the last twenty years, two cryptic species of the *B. tabaci* complex*,* Middle East-Asia Minor 1 (formerly known as the B “biotype,” hereafter MEAM1) and Mediterranean (formerly known as the Q “biotype,” hereafter MED) have invaded many regions of the world (Dalton 2006; Hu et al. 2011). They have caused serious damages to local agriculture through direct plant sap sucking and transmission of plant pathogenic viruses (Oliveira et al. 2001). What is more, the rapid invasion of MEAM1 and MED has caused the replacement of many indigenous cryptic species of the *B. tabaci* complex (Liu et al. 2007; Hu et al. 2011; Muñiz et al. 2011; Rao et al. 2011). These events provide us a unique opportunity for studying the evolution and transmission of *Wolbachia* among different *B. tabaci* cryptic species, which were geographically isolated in history but have become sympatric recently.

Previous studies have investigated the diversity of *Wolbachia* in the *B. tabaci* species complex (Nirgianaki et al. 2003; Chiel et al. 2007; Gueguen et al. 2010; Chu et al. 2011; Pan et al. 2012; Singh et al. 2012; Bing et al. 2013a). However, most of these reports focused on the two invasive cryptic species MEAM1 and MED and only used one to three marker genes in the investigation, and the distribution of *Wolbachia* in most indigenous *B. tabaci* cryptic species remains largely unknown. In this study, we examined the distribution of *Wolbachia* in *B. afer* and 10 cryptic species of the *B. tabaci* species complex collected from 13 provinces of China and one state of Australia. We report: (1) the prevalence of *Wolbachia* in *B. afer* and *B. tabaci*; (2) the discovery of a probably new *Wolbachia* (supergroup O) in whiteflies by sequencing of *rrs* gene and four protein-coding genes (*fbpA*, *hcpA*, *gltA,* and *groEL*); (3) the diversity and phylogenetic status of *Wolbachia* strains within these whiteflies; and (4) evidence for horizontal transfer of *Wolbachia* among *B. tabaci* cryptic species.

## Materials and Methods

### Whitefly collection and DNA extraction

**Figure 1:**
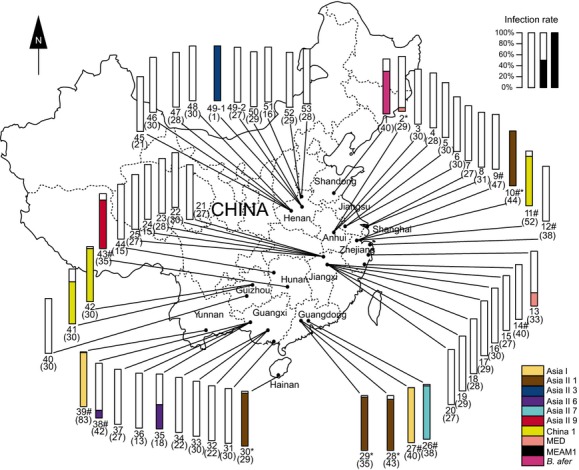
Localities of sampling and infection frequencies of *Wolbachia* in 53 Chinese populations of *Bemisia*. About 30 individuals from each population were subjected to diagnostic PCR analysis. Whiteflies collected from the same host plant in the same locality were considered as one population. The Arabic numerals correspond to populations numbered in Table A1. Figures in parentheses indicate the numbers of individuals sampled from each of the populations. Different colors represent *B. afer* and different cryptic species of the *B. tabaci* complex. The “#” signs indicate the laboratory lines that had been maintained on cotton since collection, and the “*” signs indicate the 5 populations that are positive for *Wolbachia* supergroup O (referred to in Table 3 and Fig. 2).

*Bemisia* specimens were collected from 13 provinces of China and one state of Australia. Details for collection (geographical locations, host plants, and dates) of those populations are summarized in Fig. 1 and Table A1. Whiteflies collected from the same locality and host plant were considered as one population. Whitefly samples were initially immersed in 95% ethanol after collection and subsequently kept at −20°C until DNA extraction. Total whitefly DNA was extracted from individual adult specimens according to the method of DeBarro and Driver (1997). The quality of the DNA samples was confirmed by PCR amplification of a 0.8 kb fragment of whitefly mitochondrial cytochrome oxidase I (*mtCOI*) gene using the primers C1-J-2195 and L2-N-3014 (Table A2). Cryptic species of *B. tabaci* were first identified based on the polymerase chain reaction restriction fragment length polymorphism (PCR-RFLP) method described by Qin et al. (2013), and the sex of whiteflies was identified through genital morphology. A total of 1658 whitefly DNA samples were positive for PCR amplification using the *mtCOI* primers, indicating satisfactory quality of the DNA templates.

### Diagnostic screening of *Wolbachia*

**Figure A3:**
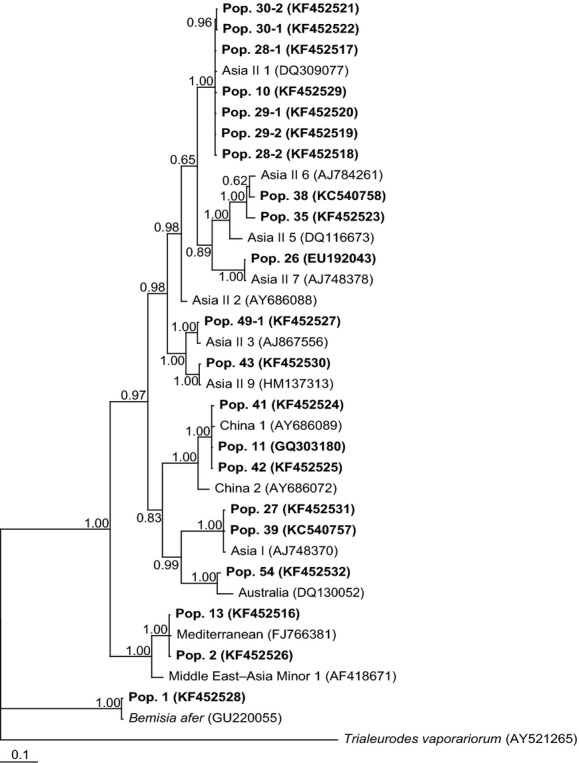
Phylogenetic analysis of the *Bemisia* spp. based on whitefly *mtCOI* gene sequences (657 sites). *Trialeurodes vaporariorum* is used as out group. Reference sequences are obtained from the CSIRO data access portal (De Barro and Boykin 2013). The tree was constructed using a TIM3 + I + G substitution model for Bayesian analysis. Bayesian posterior probabilities are indicated at nodes. The sequences obtained in this study are shown in bold. The bar indicates a branch length of 0.1 substitutions/site. The sequence GenBank accession numbers are shown in parentheses.

The presence of *Wolbachia* was screened based on the amplification of a 0.6 kb fragment with the *Wolbachia rrs* primers (Table A2). Standard PCR analyses were performed using 2×EasyTaq PCR SuperMix (TransGen, Beijing, China) in a PTC-200 Thermocycler (Bio-Rad, Hercules, CA). PCR procedures were an initial step of 94°C for 3 min, followed by 32 cycles of 94°C for 30 s, 55°C for 30 s, and 72°C for 45 s and a final step of 72°C for 10 min. Amplified DNA products were electrophoresed on agarose gels and stained with GelRed (Biotium, San Francisco, CA). To verify PCR results, amplified bands (especially uncertain ones) were purified by AxyPrep DNA gel extraction kit (Axygen, Silicon Valley, CA) and cloned into the pGEM-T vector (Promega, Madison, WI). Plasmids containing the DNA inserts of expected sizes were confirmed by PCR and sequenced in an ABI 3730 DNA analyzer (Applied Biosystems, Foster City, CA). Sequencing results were then checked by Blast in NCBI nr database (http://blast.ncbi.nlm.nih.gov/Blast.cgi). Only those individuals, which were blasted to expected products of the specific primers, were considered to be infected. All PCRs included a negative control (sterile water instead of DNA) and a positive control (DNA of China 1 whitefly). For *Wolbachia*-positive populations, *Bemisia* species were further identified by phylogenetic analysis of the *mtCOI* gene (Fig. A3). *Wolbachia* infection rates between whitefly sexes were statistically tested using the Fisher's exact test. Statistical significance of the infection rates among different *B. afer* and *B. tabaci* cryptic species was calculated using the *χ*^2^ test and corrected by the Bonferroni procedure. All statistical analyses were performed using the Data Processing System (DPS) software (Tang and Zhang 2013).

### Sequencing and typing of *Wolbachia*

As the phylogenetic analysis of the 0.6 kb *rrs* sequences indicated a possible new supergroup of *Wolbachia*, we amplified the *rrs* gene from whitefly populations and introduced an endonuclease *Vsp*I (AT/TAAT) (Fisher Scientific, Pittsburgh, PA) to digest the target bands, to investigate the infection prevalence of the new supergroup of *Wolbachia*. Several whitefly individuals were then randomly selected for sequencing confirmation of PCR-RFLP results. The *groEL*, *wsp,* and MLST (*gatB*, *coxA*, *hcpA, fbpA,* and *ftsZ*) genes from every combination of host species and *rrs* genotype were amplified by TransTaq-T DNA Polymerase (TransGen), cloned into pEASY-T1 vectors (TransGen) and sequenced on ABI 3730 DNA analyzer (Applied Biosystems).

In addition, to confirm the finding of the new *Wolbachia* supergroup, nearly a complete *rrs* sequence (1417 bp) and a partial *gltA* sequence (659 bp) were amplified from one *Wolbachia* new supergroup singular-infected population. PCR amplifications, DNA cloning and sequencing procedures were accomplished as described previously. The cycling procedures were the same as described earlier with changes on the annealing temperature for different primers. Primer sequences and annealing temperatures of *rrs*, *gltA*, *groEL*, *wsp,* and the MLST genes were listed in Table A2.

Sequences of MLST and *wsp* genes were manually trimmed in line with the template provided in *Wolbachia* MLST website and compared with sequences in the *Wolbachia* MLST database (http://pubmlst.org/wolbachia/). Novel sequences were submitted to the database curators as new alleles. Each unique combination of five MLST sequences was designated a strain type (ST) number in the *Wolbachia* MLST database (Baldo et al. 2006). Previously published sequences from other whiteflies were added to the data set to increase the power of statistical comparisons. All newly obtained allele numbers and ST numbers in this study are summarized in Table 1.

**Table 1:** GenBank accession numbers of *Wolbachia* sequences and allele profiles of *Wolbachia-*positive whitefly populations

Pop.	Strain	Species	*rrs*	*groEL*	ST	*gatB*	*coxA*	*hcpA*	*ftsZ*	*fbpA*	*wsp*	HRV1	HRV2	HRV3	HRV4
1	*wBa*_1	*Bemisia afer*	KF454753	KF452533	**382**	KF452586 (105)	KF452567 (11)	KF454725 **(220)**	KF452573 (11)	KF454737 (162)	KF465800 (**670**)	**(231)**	**(265)**	(143)	(23)
2	*wBt*_2	MED	KF454762	–		–	–	–	–	KF454744 **(386)**	KF465817 (**668**)	**(230)**	**(264)**	(3)	(23)
10	*wBt*_10	Asia II 1	KF454771	KF452543	**391**	KF452588 **(207)**	KF452561 (88)	KF454726 **(234)**	KF452576 **(170)**	KF454746 (**390**)					
11	*wBt*_11	China 1	JF795502	KF452544	**379**	KF452591 (105)	KF452563 (88)	KF454731 (13)	KF452578 (170)	KF454752 (9)	KF465816 **(669)**	(2)	(17)	(3)	(2)
13	*wBt*_13	MED	KF454764	–		–	–	KF454719 **(236)**	KF452575 **(181)**	KF454745 (387)	KF465805 (**671**)	**(232)**	(17)	(3)	(2)
26	*wBt*_26	Asia II 7	JF795503	KF452536	**378**	KF452590 (105)	KF452562 (88)	KF454730 (106)	KF452577 (7)	KF454747 (387)	KF465815 **(665)**	(78)	(88)	(90)	(2)
27	*wBt*_27	Asia I	KF454755	KF452540	**385**	KF452594 (**210**)	KF452565 (88)	KF454721 (106)	KF452580 (7)	KF454751 **(387)**	KF465810 **(**163**)**	(78)	(88)	(90)	(23)
28-1	*wBt*_28-1	Asia II 1	KF454768	KF452534	**392**	KF452596 (207)	KF452560 (88)	KF454727 **(230)**	KF452581 (170)	KF454734(**392**)	KF465806 (669)	(2)	(17)	(3)	(2)
28-2	*wBt*_28-2	Asia II 1	KF454769	KF465814		–	KF452558 (88)	KF454728 **(231)**	–	KF454733 (390)	–				
29-1	*wBt*_29-1	Asia II 1	KF454758	KF452535	**390**	KF452595 (207)	KF452559 (88)	KF454729 **(232)**	KF452582 (170)	KF454738 (9)	KF465807 (669)	(2)	(17)	(3)	(2)
29-2	*wBt*_29-2	Asia II 1	KF454757	KF452547		–	–	–	–	–	–				
30-1	*wBt*_30-1	Asia II 1	KF454759	KF452538	**389**	KF452600 **(208)**	KF452551 (88)	KF454715 (106)	KF452584 (170)	KF454735 **(393**)	KF465808 (669)	(2)	(17)	(3)	(2)
30-2	*wBt*_30-2	Asia II 1	KF454760	KF452539		–	KF452552 (88)	KF454716 (13)		KF454736 **(391)**	KF465809 (669)	(2)	(17)	(3)	(2)
35	*wBt*_35	Asia II 6	KF454761	KF452601	**393**	KF452599 **(216)**	KF452553 (88)	KF454722 (106)	KF452570 (7)	KF454741 (387)	KF465818 (163)	(78)	(88)	(90)	(23)
38	*wBt*_38	Asia II 6	KF454767	KF452537	**394**	KF452589 (207)	KF452550 (88)	KF471409 **(235)**	KF452569 (170)	KF454740 (9)	KF465812 (669)	(2)	(17)	(3)	(2)
39	*wBt*_39	Asia I	KF454766	KF452541	**395**	KF452587 (207)	KF452556 (88)	KF454718 (106)	KF452568 **(182)**	KF454750 (387)	KF465811 **(672)**	(2)	(17)	**(261)**	(2)
41	*wBt*_41	China 1	KF454765	KF452548	**377**	KF452597 (207)	KF452557 (88)	KF454724 (13)	KF452572 (170)	KF454743 (9)	KF465803 (669)	(2)	(17)	(3)	(2)
42	*wBt*_42	China 1	KF454770	KF452546	**383**	KF452598 **(209)**	KF452554 (88)	KF454723 (13)	KF452571 (170)	KF454742 (9)	KF465802 (669)	(2)	(17)	(3)	(2)
43	*wBt*_43	Asia II 9	KF454756	KF452542	**384**	KF452593 (207)	KF452566 (88)	KF454720 (13)	KF452583 (170)	KF454748 (386)	KF465813 (160)	(2)	(17)	(3)	(23)
49-1	*wBt*_49-1	Asia II 3	KF454763	KF452549	**396**	KF452585 (207)	KF452555 (88)	KF454717 **(228)**	KF452574 **(180)**	KF454739 (386)	KF465804 (160)	(2)	(17)	(3)	(23)
54	*wBt*_54	Australia	KF454754	KF452545	**380**	KF452592 (207)	KF452564 (88)	KF454732 **(221)**	KF452579 (170)	KF454749 (9)	KF465801 (160)	(2)	(17)	(3)	(23)

ST numbers and allele profile numbers in bold represent new sequence data generated from this study.

### Molecular phylogenetic analysis

Phylogenetic analyses were constructed using (1) *Wolbachia* sequences of *rrs*, *gltA*, *groEL,* MLST, and *wsp* genes of different supergroups from various hosts; and (2) whitefly *mtCOI* sequences. All sequences used in this study were edited and aligned manually using Clustal W (ver. 1.6) (Thompson et al. 1994) in MEGA (ver. 5.10) (Tamura et al. 2011). The Gblocks program (ver. 0.91b) (Castresana 2000) was used to remove poorly aligned positions and to obtain nonambiguous sequence alignments. The best-fit evolutionary model for the sequence data was determined using hierarchical likelihood ratio tests and Akaike information criterion with the program jModelTest (ver. 0.1.1) (Posada 2008). Phylogenetic trees were constructed with the Bayesian inference using MrBayes (ver 3.1.2) (Ronquist and Huelsenbeck 2003). For these gene data, 5 million generations were run; 50,000 trees were obtained, and the first 25% trees were discarded as burn-in. The resulting phylogenetic trees were visualized in TreeView (ver. 1.6.6) (Page 1996). The comparison between the phylogeny of whitefly *mtCOI* and *Wolbachia* concatenate MLST data sets was constructed with Dendroscope (ver. 3.2.8) (Huson and Scornavacca 2012). The new *Wolbachia rrs*, *gltA*, *groEL*, *wsp,* and MLST gene sequences and whitefly *mtCOI* gene sequences have been deposited in the GenBank database (Table 1, Fig. A3 and Table A4).

The pairwise genetic divergence of different *Wolbachia* supergroups was calculated using the Kimura 2-parameter method (Kimura 1980) in MEGA (ver. 5.10) (Tamura et al. 2011). Because recombination of sequences has potentially disruptive influences on phylogenetic-based molecular evolution analyses (Martin et al. 2011), alignments of individual and concatenated genes were checked for significant levels of recombination using the Phi test (Bruen et al. 2006) in SplitsTree4 under default conditions (Huson and Bryant 2006). When recombination was tested to be significant, a phylogenetic network framework was constructed based on uncorrected P distances using the Neighbor-net method (Bryant and Moulton 2004) implemented in SplitsTree4 (ver. 4.13.1) (Huson and Bryant 2006).

### FISH

Localization of *Portiera* and *Wolbachia* was studied in nymphs and adults of *B. tabaci* Asia II 1 (Pop. 10) and Asia II 9 (Pop. 43) using fluorescence labeled probes specifically targeting the *rrs* genes of these bacteria. We followed the previous protocols for the FISH experiments (Bing et al. 2013b). Briefly, specimens were collected directly into Carnoy's fixative and fixed overnight. After fixation, the samples were hybridized overnight in hybridization buffer (20 mM Tris-HCl, pH 8.0, 0.9 M NaCl, 0.01% sodium dodecyl sulfate, 30% deionized formamide) containing 10 pmol of fluorescent probes. The probe, BTP1-Cy3 (5′-Cy3- TGTCAGTGTCAGCCCAGAAG-3′), was used to target *rrs* gene of *Portiera* (Gottlieb et al. 2006). A new probe, Wolb-1-488 (5′- Alexa Fluor 488- TAATATAGGCTCATCTAATAGCAA -3′), was designed to target *rrs* gene of *Wolbachia*. The specificity of the detection was first checked by “probe match” in RDP 10 (update to May 14, 2013) (Cole et al. 2009) and BLAST in nr database of NCBI (http://blast.ncbi.nlm.nih.gov/Blast.cgi) and then confirmed using the following controls: a no probe control and *Wolbachia*-free whiteflies (samples of the *B. tabaci* MED species and MEAM1 species). Stained samples were wholly mounted and viewed under a Zeiss LSM780 confocal microscope.

## Results

### Prevalence of *Wolbachia* in *Bemisia* species

*Bemisia tabaci* has a wide distribution in China. In this study, samples of *Bemisia* were obtained from 24 localities of 13 provinces of this country (Fig. 1). In all, these samples represent one population of *B. afer* and 52 populations of 9 cryptic species of the *B. tabaci* complex from China (Table A1). In addition, one sample of an indigenous species of the *B. tabaci* complex was obtained from Australia (Table A1).

Of the 1658 individuals examined, *rrs* PCR assays indicated that the infection rates of *Wolbachia* varied among species, and even among populations of a given species, ranging from 0% to 100% (Fig. 1; Table 2, Chi-square test, *P* < 0.0001), but did not differ between sexes in each of the populations that were tested statistically (Table A1). The incidence of *Wolbachia* infection in indigenous whiteflies (79.61%, *n* = 618) was significantly higher than that in invasive whiteflies (MEAM1 and MED, 0.96%, *n* = 1040; Fisher's exact 2-tailed test, *P* < 0.0001). The infection rate of *Wolbachia* also varied among different indigenous species of the *B. tabaci* complex. For instance, 88.4% of China 1 whiteflies were positive for *Wolbachia* while so were only 2.1% of Asia II 3 whiteflies (Table 2). The rate of *Wolbachia* infection in *B. afer* was 77.5% (Table 2).

**Table 2:** Rates of *Wolbachia* infection in *Bemisia afer* and cryptic species of the *B. tabaci* complex

Whitefly species	*n*	No. of localities	Infection rate (%)^1^
*B. afer*	40	1	77.5 a
Asia I	123	2	99.2 b
Asia II 1	151	4	96.7 bc
Asia II 3	48	2	2.1 de
Asia II 6	60	2	23.3 d
Asia II 7	38	1	97.4 ab
Asia II 9	35	1	88.6 ab
China 1	112	3	88.4 ac
Australia	11	1	100.0 ab
MEAM1	334	12	0.0 e
MED	706	26	1.4 e

1 Figures followed by different letters differ significantly (adjusted *P* < 0.05, using Bonferroni corrections).

### Diversity of *Wolbachia* infections

**Figure 2:**
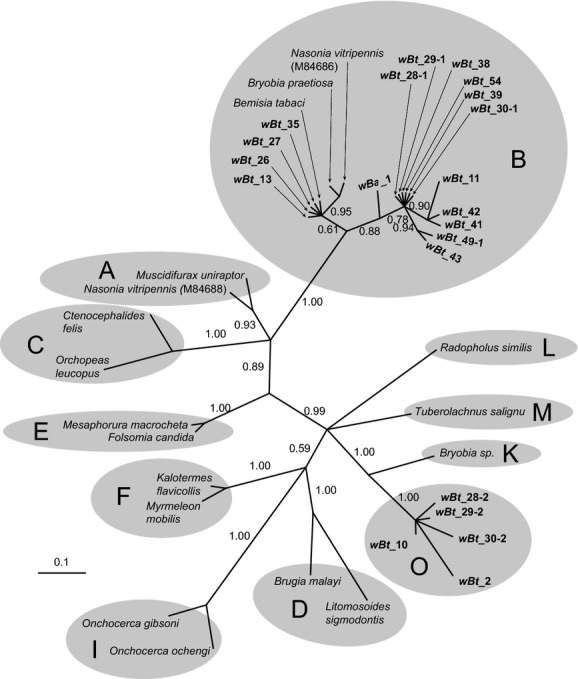
Phylogeny of the *Wolbachia* identified from *Bemisia afer* and cryptic species of the *B. tabaci* complex based on bacterial *rrs* gene sequences (592 sites). *Wolbachia* strains are characterized by the names of their host species. The tree was constructed using a TPM1uf + G substitution model for Bayesian analysis. Bayesian posterior probabilities are shown on the branches. Sequences obtained in this study are shown in bold. The bar indicates a branch length of 0.1 substitutions/site. The sequence names and GenBank accession numbers are listed in Tables A4 and A6.

For those *Wolbachia*-positive populations, 2–3 individuals were further analyzed by sequencing *Wolbachia rrs* gene (592 bp) and performing Bayesian phylogenetic analysis. Most of whitefly *Wolbachia rrs* sequences were clustered into the supergroup B (Fig. 2). However, several *Wolbachia rrs* sequences obtained from four Asia II 1 populations (*wBt*_10, *wBt*_28-2, *wBt*_29-2, *wBt*_30-2) and one MED population (*wBt*_2) formed a strict and robust monophyletic clade (Fig. 2).

### Identification of *Wolbachia* supergroup O

**Figure A5:**
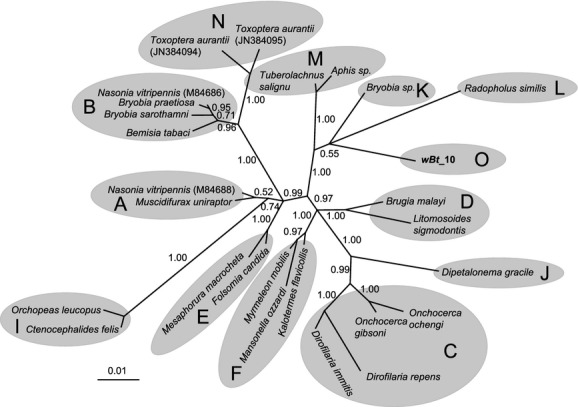
Phylogenetic position of the *Wolbachia* identified from *Bemisia tabaci* putative species Asia II 1 (*wBt*_10) based on bacterial *rrs* gene sequences (1317 sites). *Wolbachia* strains are characterized by the names of their host species. The tree was constructed using a HKY + G substitution model for Bayesian analysis. Bayesian posterior probabilities are shown on the branches. The sequence obtained in this study is shown in bold. The bar indicates a branch length of 0.01 substitutions/site. The names and sequence GenBank accession numbers are listed in Tables A4 and A6.

Further Bayesian phylogenetic analysis on a nearly complete *rrs* sequence (1317 bp) of the strange *Wolbachia* (*wBt*_10) revealed that this *Wolbachia* differed widely from other known *Wolbachia* (Fig. A5). The divergence of *rrs* between *wBt*_10 and the supergroup M is 2.52%, which is the smallest of that between *wBt*_10 and all previously reported *Wolbachia* supergroups (A to N) (Table A6). In view of these apparent differences, we proposed to name these *Wolbachia* as supergroup O temporarily.

**Figure A7:**
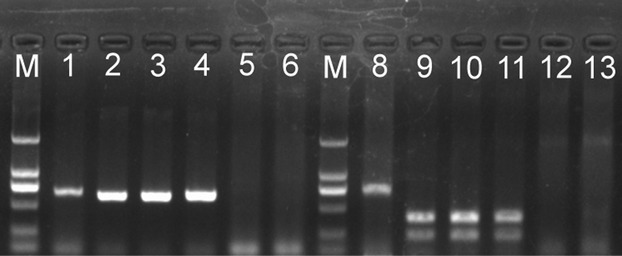
RFLP pattern of PCR products of *rrs* gene of the *Wolbachia* supergroup O and B in *Bemisia tabaci* corresponding to *Vsp*I (*Ase*I) digestion. The different profiles were obtained from individuals representing different *Wolbachia* in *B. tabaci*. The bands shown on the bottom are primer dimers. Lane 1, undigested *Wolbachia* O obtained by PCR from Asia II 1; lane 2–4, undigested *Wolbachia* B obtained by PCR from Asia II 7, Asia I and China 1, respectively; lane 5–6, *Wolbachia* PCR amplified production from MEAM1 and MED as controls; lane 8, digested *Wolbachia* O obtained by PCR from Asia II 1; lane 9–11, *Wolbachia* B obtained by PCR from Asia II 7, Asia I and China 1, respectively; lane 12–13, *Wolbachia* PCR amplified production from MEAM1 and MED as controls; lane M, DNA size markers (100, 250, 500, 750, 1000, and 2000 bp from bottom to top).

Our results thus showed the presence of two genetically distant *Wolbachia* supergroups in whiteflies. To clarify the detailed infection prevalence of *Wolbachia* O in *Bemisia* whiteflies, PCR-RFLP was introduced to digest all positive *rrs* PCR products by the restriction enzymes *Vsp*I. No *Vsp*I restriction site was found in *rrs* sequences of *Wolbachia* supergroup O, whereas *rrs* amplicons from *Wolbachia* supergroup B could be digested into multiple bands by *Vsp*I (Fig. A7). Whiteflies in populations 2 (MED species) and 10 (Asia II 1 species) are infected singly by *Wolbachia* O, whereas populations 28, 29, and 30 (all three are Asia II 1 species) are infected by both *Wolbachia* O and *Wolbachia* B (Table 3).

**Table 3:** Infection frequencies of the *Wolbachia* O in five populations of the *Bemisia tabaci* complex

				Single infection (%)		
					
Pop. no.	Cryptic species	*n*^1^	% without *Wolbachia* infection	O	B	Double infection (%)
2	MED	29	93.1	6.9		
10	Asia II 1	44		100		
28	Asia II 1	43	6.9	14.0	62.8	16.3
29	Asia II 1	35	2.9	5.7	65.7	25.7
30	Asia II 1	29	3.4	24.1	3.4	69.0

1 Number of whitefly individuals collected from the five populations shown with asterisks in Fig. 1 and Table A1.

**Figure 3:**
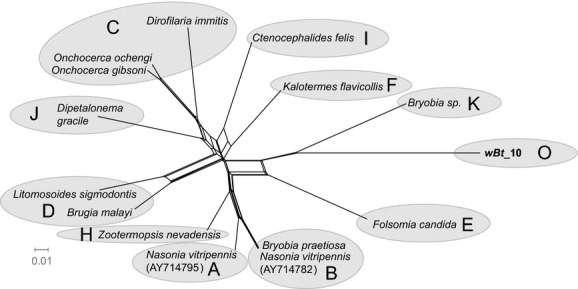
Phylogenetic position of the *Wolbachia* identified from the putative species Asia II 1 of the *Bemisia tabaci* complex based on bacterial *gltA* gene sequences (636 sites) using the Neighbor-net method. Each edge (or a set of parallel edges) corresponds to a split in the data set and has a length equal to the weight of the split. The sequence obtained in this study is shown in bold. The bar indicates a branch length of 0.1 substitutions/site. The names and sequence GenBank accession numbers are listed in Table A4.

**Figure 4:**
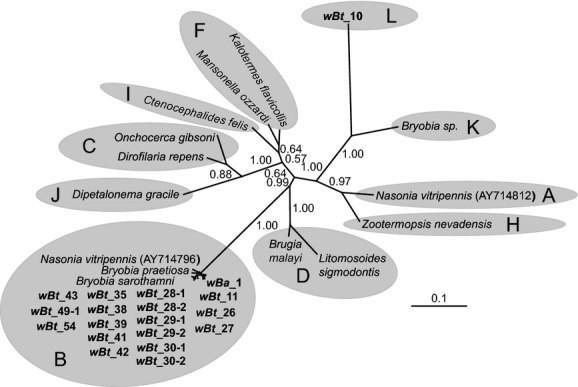
Phylogenetic position of the *Wolbachia* identified from *Bemisia afer* and *B. tabaci* putative species based on bacterial *groEL* gene sequences (491 sites). *Wolbachia* strains are characterized by the names of their host species. The tree was constructed using a GTR + G substitution model for Bayesian analysis. Bayesian posterior probabilities are shown on the branches. The sequence obtained in this study is shown in bold. The bar indicates a branch length of 0.1 substitutions/site. The names and sequence GenBank accession numbers are listed in Table 1 and Table A4.

At least one of the eight tested protein-coding genes (*gltA*, *groEL*, MLST (*gatB*, *coxA*, *hcpA*, *fbpA*, *ftsZ*), and *wsp* genes) was successfully amplified and sequenced for all *Wolbachia-*infected populations in this study. Both neighbor-net analysis of *fbpA*, *gltA*, *hcpA* gene and Bayesian interference of *groEL* gene supported the existence of the *Wolbachia* supergroup O (*wBt*_10) (Figs. 3 and 4, and Figs. A10 and A12). However, it should be noted that the results of phylogenetic analyses with different genes were not always consistent. In particular, analysis of *fbpA*, *groEL*, and *hcpA* gene clustered some strange *Wolbachia*, such as *wBt*_29-2 and *wBt*_30-2, which were identified as O by *rrs* gene, into supergroup B (Fig. 4, and Figs. A10 and A12).

**Figure 5:**
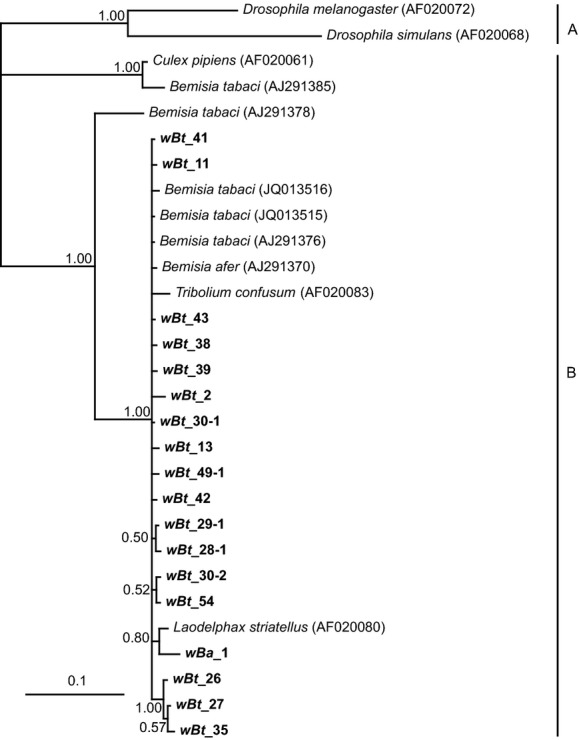
Phylogenetic position of the *Wolbachia* identified from *Bemisia afer* and *B. tabaci* putative species based on bacterial *wsp* gene sequences (512 sites). *Wolbachia* strains are characterized by the names of their host species. The two *Drosophila wsp* sequences are the outgroups. The tree was constructed using a TIM3 + G substitution model for Bayesian analysis. Bayesian posterior probabilities are shown on the branches. The sequence obtained in this study is shown in bold. The bar indicates a branch length of 0.1 substitutions/site. The names and sequence GenBank accession numbers are listed in parentheses and Table 1.

**Figure A8:**
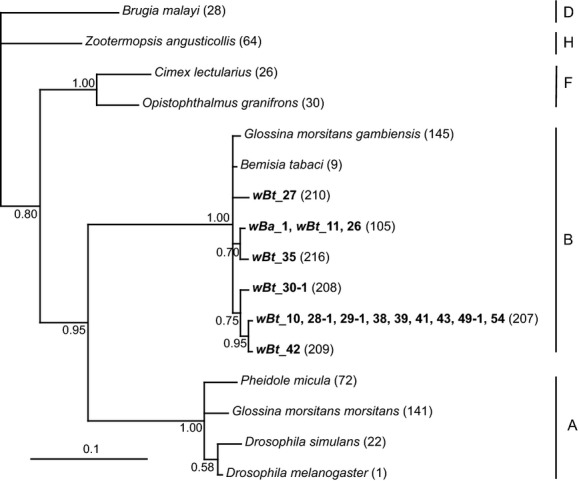
Phylogenetic position of the *Wolbachia* identified from *Bemisia afer* and *B. tabaci* putative species based on bacterial *gatB* gene sequences (369 sites). *Wolbachia* strains are characterized by the names of their host species and allele numbers from the MLST database. The tree was constructed using a TPM2uf + G substitution model for Bayesian analysis. Bayesian posterior probabilities are shown on the branches. The sequences obtained in this study are shown in bold. The bar indicates a branch length of 0.1 substitutions/site.

**Figure A9:**
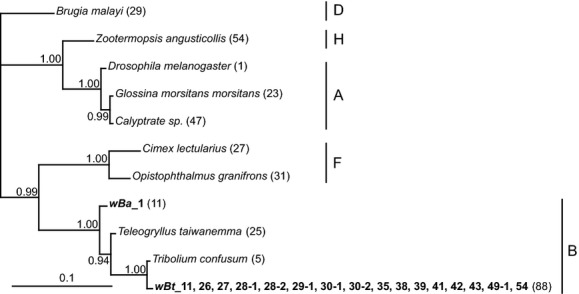
Phylogenetic position of the *Wolbachia* identified from *Bemisia afer* and *B. tabaci* putative species based on bacterial *coxA* gene sequences (402 sites). *Wolbachia* strains are characterized by the names of their host species and allele numbers from the MLST database. The tree was constructed using a TIM1 + G substitution model for Bayesian analysis. Bayesian posterior probabilities are shown on the branches. The sequences obtained in this study are shown in bold. The bar indicates a branch length of 0.1 substitutions/site.

**Figure A10:**
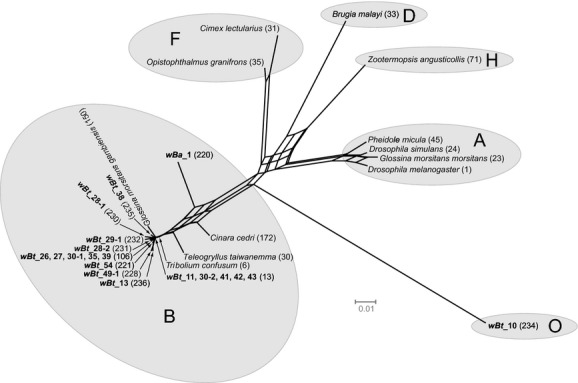
Phylogenetic position of the *Wolbachia* identified from *Bemisia afer* and *B. tabaci* putative species based on bacterial *hcpA* gene sequences (444 sites) using the Neighbor-net method. Each edge (or a set of parallel edges) corresponds to a split in the data set and has length equal to the weight of the split. The sequence obtained in this study is shown in bold. MLST Database allele numbers of *hcpA* sequences are shown in parenthesis. The bar indicates a branch length of 0.01 substitutions/site.

**Figure A11:**
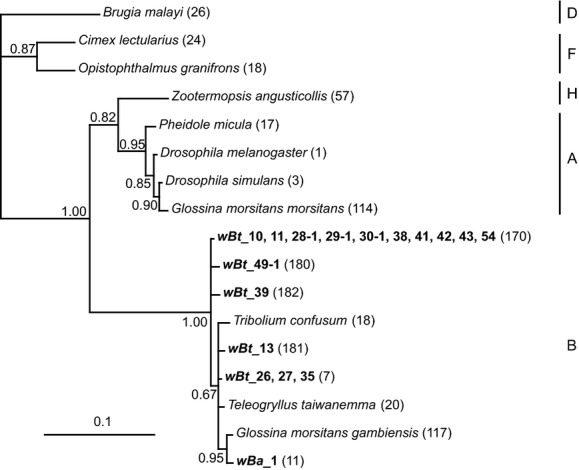
Phylogenetic position of the *Wolbachia* identified from *Bemisia afer* and *B. tabaci* putative species based on bacterial *ftsZ* gene sequences (435 sites). *Wolbachia* strains are characterized by the names of their host species and allele numbers from the MLST database. The tree was constructed using a TrN + I substitution model for Bayesian analysis. Bayesian posterior probabilities are shown on the branches. The sequences obtained in this study are shown in bold. The bar indicates a branch length of 0.1 substitutions/site.

**Figure A12:**
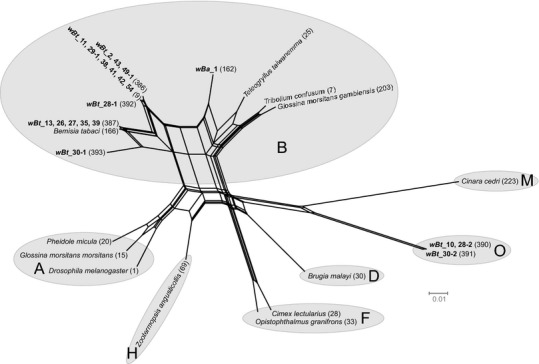
Phylogenetic position of the *Wolbachia* identified from *Bemisia afer* and *B. tabaci* putative species based on bacterial *fbpA* gene sequences (429 sites) using the Neighbor-net method. Each edge (or a set of parallel edges) corresponds to a split in the data set and has length equal to the weight of the split. The sequences obtained in this study are shown in bold. MLST Database allele numbers of *fbpA* sequences are shown in parenthesis. The bar indicates a branch length of 0.01 substitutions/site.

Sixteen STs were identified in whiteflies from this study, and all of them are new to the MLST database (Table 1). Though efforts were made, some PCRs failed when amplifying the MLST and *wsp* genes from supergroup O-infected whiteflies (Table 1). As a result, sequences from supergroup O-infected whiteflies were excluded from phylogenetic analysis of the concatenated MLST sequences. Respective Bayesian interference of separate *gatB*, *coxA*, *ftsZ,* and *wsp* genes showed that all *Wolbachia* detected in whiteflies belonged to supergroup B (Fig. 5, and Figs. A8, A9 and A11). Neighbor-net analysis clustered the majority of *Wolbachia* into supergroup B except for *wBt*_10 (Figs. A10 and A12). The *hcpA* genes from *wBt*_10 and *fbpA* from *wBt*_10 and *wBt*_28-2 formed a separate branch that differs distinctly from all known reference sequences.

### Co-divergence between the divergence of *Wolbachia* supergroup B and whitefly species

**Figure 6:**
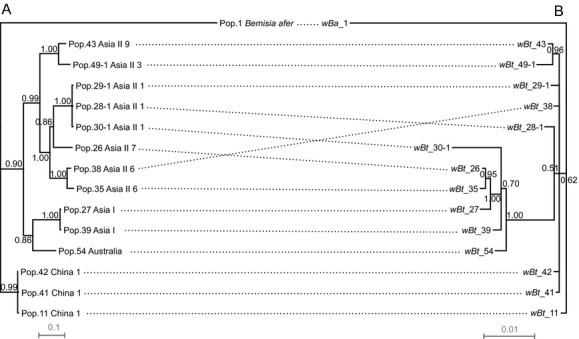
Comparisons of *Bemisia* and *Wolbachia* phylogenies. A, the whitefly phylogeny constructed based on Bayesian analysis of *mtCOI* sequences (817 bp) as shown in Fig. A3 using TIM3 + I + G model. B, the *Wolbachia* phylogeny constructed based on Bayesian analysis of concatenated sequences of MLST genes (2079 bp) as shown in Table 1 using GTR + I + G model. Bayesian posterior probabilities are shown on the branches. Dashed lines connect hosts to their respective *Wolbachia* strains. The scale bar is in units of substitutions/site.

The codivergence of *Bemisia* and *Wolbachia* supergroup B was assessed by studying the sequences of partial *mtCOI* gene and *Wolbachia* MLST genes. For those *Wolbachia* identified from *B. tabaci*, very poor congruence was found between the phylogenies of *mtCOI* and concatenated MLST genes (Fig. 6). The topology of MLST tree differs obviously from that of *mtCOI*. Whiteflies belonging to the same cryptic species harbored distant *Wolbachia* strains. For instance, two populations (*wBt*_35 and *wBt*_38) of *Wolbachia* identified from Asia II 6 are clustered in different phylogenetic groups.

### Localization of *Wolbachia* in *Bemisia tabaci*

**Figure 7:**
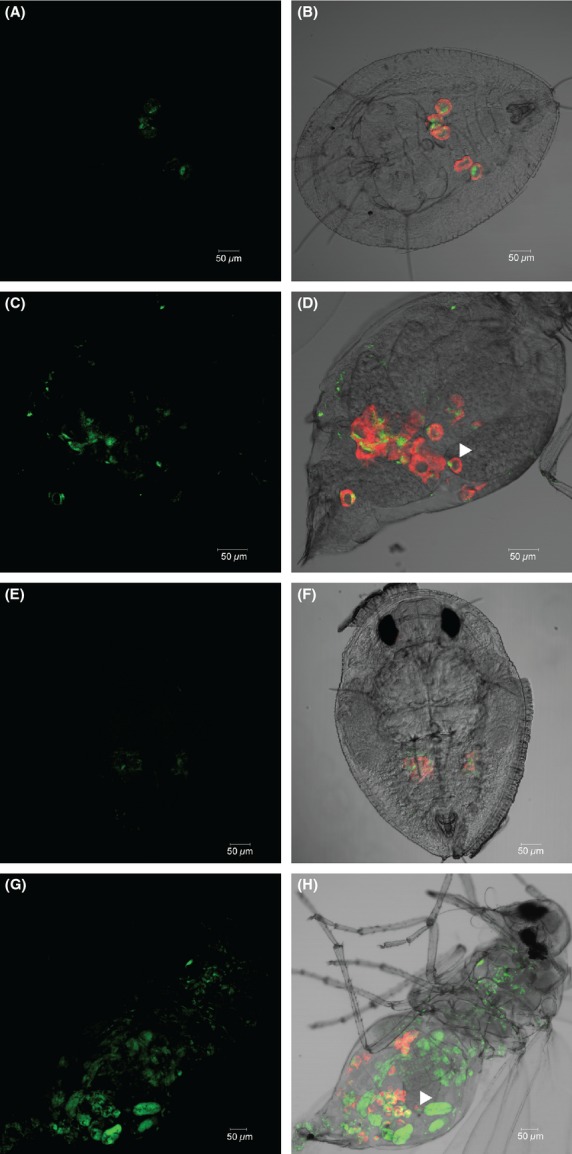
Whole-mount FISH of *Bemisia tabaci* nymphs and female adults using a *Portiera*-specific probe (red) and a *Wolbachia*-specific probe (green). Upper column, Asia II 1 nymph and female adult; lower column, Asia II 9 nymph and female adult. A, C, E, G: *Wolbachia* channel on a dark-field channel. B, D, F, H: Overlay of *Portiera* and *Wolbachia* channels on a bright-field channel. White triangles in D and H indicate anterior poles of the oocytes. Signals on legs, joints, and wings are chitin autofluoresence.

The FISH of bacteria revealed that *Portiera* was seen exclusively in the bacteriocytes of whiteflies. In the tested nymphs, *Wolbachia* was strictly located in the bacteriocytes among the abundant *Portiera* (Fig. 7). Nevertheless, in the adults, *Wolbachia* was detected both outside and inside the bacteriocytes (Fig. 7). Signals of *Wolbachia* shown at the anterior pole of the oocytes of female adults indicate its vertical transmission (arrows marked in Fig. 7 D & H).

## Discussion

*Wolbachia* is widely distributed among invertebrates and is considered as the most prevalent symbiont identified so far. Though several research groups have investigated the prevalence of *Wolbachia* in some cryptic species of the *B. tabaci* complex*,* our study represents the first comprehensive analysis of *Wolbachia* infection among both invasive and indigenous cryptic species of the *B. tabaci* complex in Asia. In addition, compared with previous investigations, we used five more molecular markers in our analyses.

### Prevalence of *Wolbachia* varies between invasive and indigenous whiteflies

In this study, *Wolbachia* infection rates in five (Asia I, Asia II 1, Asia II 7, Asia II 9, and China 1) of the seven Chinese indigenous species reached over 70%. In contrast, *Wolbachia* infection rate in the MED populations from China was only 1.4% (10/706), and no infection (0/334) was detected in all MEAM1 populations from this country. The low rates of *Wolbachia* infection in MEAM1 and MED agree with those observed in most previous studies. For example, in populations of MEAM1 and MED from Europe and Western Africa, infection rates of *Wolbachia* varied from 0–8.3% and 0–33% (Nirgianaki et al. 2003; Chiel et al. 2007; Gueguen et al. 2010; Skaljac et al. 2010; Chu et al. 2011; Thierry et al. 2011; GnankinÉ et al. 2013). And in populations of MEAM1 and MED from China, the rates of *Wolbachia* infection were 0.2% (1/456) and 0% (0/1149), respectively (Pan et al. 2012). As a whole, our data indicate a high variability of prevalence of *Wolbachia* between cryptic species of the *B. tabaci* complex. In our sampling, we obtained adequate numbers of whitefly individuals for five (Asia II 1, Asia II 6, China 1, MEAM1, and MED) of the 11 whitefly species from both laboratory and field. The data indicate that the frequencies of *Wolbachia* infection between laboratory and field populations in each of the five species appeared similar (Table A1). Thus, the laboratory rearing seemed to have exerted little effects on the frequencies of *Wolbachia* infection in these whitefly species. Until now, factors underlying the high variability of *Wolbachia* infection between the whitefly species are virtually unknown but certainly warrant future investigations.

In contrast to a previous study that reports absence of *Wolbachia* infection in *B. afer* populations from China (Chu et al. 2010), the rate of *Wolbachia* infection in the *B. afer* population examined in the current study reached 77.5%. Phylogenetic analysis of *rrs*, *groEL*, MLST, and *wsp* genes showed that the *Wolbachia* detected from *B. afer* belongs to supergroup B, which agrees with the report of Nirgianaki et al. (2003).

### Identification of a novel *Wolbachia* supergroup O

Preliminary Bayesian phylogenetic analysis based on *rrs* gene sequences strongly supports the existence of one strange monophyletic group compared with the other *Wolbachia* identified in whiteflies. The *rrs* sequences from five of the whitefly populations (*wBt*_2, *wBt*_10, *wBt*_28-2, *wBt*_29-2, and *wBt*_30-2) were clustered into group O. Average distance among those strange *rrs* sequences (592 bp) are 0.48%. The divergence of *rrs* between *wBt*_10 and all previously described *Wolbachia* supergroups (A to N) is higher than the 2% distance, a level of divergence that may merit the establishment of a new supergroup (Stouthamer et al. 1993; Augustinos et al. 2011). What is more, independent Bayesian analysis of *rrs* and *groEL* gene sequences and Neighbor-net analysis of *gltA, hcpA,* and *fbpA* gene sequences confirmed the distinct phylogenetic position of *wBt*_10 from the other supergroups. Based on the evidence, we propose the strange *Wolbachia* group as a new supergroup – Supergroup O.

### All previously known *Wolbachia* in *Bemisia tabaci* belong to supergroup B

Except for the five supergroup O strains, phylogenetic analysis of eight molecular markers (*rrs*, *groEL*, *gatB*, *coxA*, *hcpA*, *fbpA*, *ftsZ*, and *wsp* genes) showed that all the *Wolbachia* strains detected from Chinese whiteflies as well as one strain from the Australia species belong to supergroup B. This is consistent with previous diversity studies on *Bemisia* and *Trialeurodes* whiteflies (Nirgianaki et al. 2003; Sintupachee et al. 2006; Gueguen et al. 2010; Singh et al. 2012; Tsagkarakou et al. 2012).

### The protein-coding genes are limited in *Wolbachia* diversity investigation

At the early stage of *Wolbachia* research, the identification of *Wolbachia* strains was inferred based on the *rrs* gene (O'Neill et al. 1992; Stouthamer et al. 1993; Dumler and Walker 2005). As the research progressed, the *rrs* gene was found too conserved for further analysis of the *Wolbachia* genus. Subsequently, additional protein-coding genes (*gltA*, *groEL*, *ftsZ,* and *wsp* genes) were developed for infection and evolutionary analysis of *Wolbachia* (Werren et al. 1995b; Zhou et al. 1998; Lo et al. 2002, 2007; Casiraghi et al. 2005). Baldo et al. (2006) developed a standard MLST-based system (*gatB*, *coxA*, *hcpA*, *ftsZ*, and *fbpA*) for genotyping and strain classification of *Wolbachia* infections. However, with more exploration of *Wolbachia* diversity, conflict results occurred among these different markers (Augustinos et al. 2011). In this study, the presence of supergroup O was confirmed by *rrs* and four protein-coding genes (*fbpA*, *gltA*, *groEL*, and *hcp* genes) (Figs. 2–4, and Figs. A10 and A12). Whereas phylogenetic analysis of several protein-coding genes (*coxA*, *groEL, gatB, ftsZ,* and *wsp*) clustered many *Wolbachia* O strains into supergroup B (Figs. 4 and 5, and Figs. A9, A8 and A11). Similar phenomena have been noticed in previous studies. For example, even though the supergroup M and N have been identified as new groups of *Wolbachia* by *rrs* gene clustering, Augustinos et al. (2011) found that several popular protein-coding sequences such as *gltA*, *groEL,* and MLST genes clustered some individuals of those new groups into the old supergroup B. Besides, failures of amplifying MLST and *wsp* genes in many *Wolbachia* O-infected whiteflies (Table 1) indicated protein-coding genes may not be sufficient for investigating the diversity of *Wolbachia* in *B. tabaci*. The failure of amplification of *ftsZ* and *wsp* genes were also observed in *Wolbachia*-infected aphids (Augustinos et al. 2011). Consequently, it seems clear that phylogenetic analysis merely using protein-coding genes may underestimate the diversity of *Wolbachia*.

That inadequacy of protein-coding genes for analyzing the diversity of *Wolbachia* may be explained by: (1) primers of protein-coding genes are designed based on the earliest known *Wolbachia* (mostly A and B); and (2) different protein-coding genes suffer different selective pressure and thus have different evolutionary patterns. The *rrs* gene sequence is more conserved than *wsp* gene and the results of amplification are more stable compared with that of *wsp* or *ftsZ* genes which often produces unexpected bands (not target-size bands or not the gene of *Wolbachia*). In fact, no single pair of primers can ensure detection of all *Wolbachia* specifically among various samples (SimÕEs et al. 2011). In view of the limitation of the various primers, we suggest that infection data obtained by any of these genes should be confirmed by vector cloning and sequencing of all representative bands.

### *Wolbachia* in *Bemisia tabaci* are transmitted horizontally

Our FISH data indicate that *Wolbachia* can be vertically transmitted in whiteflies (Fig. 7), a result in agreement with that of a previous report (Gottlieb et al. 2008). In addition, our FISH data show the distribution of *Wolbachia* outside of bacteriocytes of the whitefly adults and thus also indicate potential horizontal transmission of *Wolbachia*. Not surprisingly, incongruence was found between the phylogeny of *Bemisia mtCOI* sequences and that of *Wolbachia* supergroup B based on concatenated MLST sequences (Fig. 6). In addition, in several cases, a population of a given whitefly species harbored divergent *Wolbachia* strains (e.g., Population of 28 in Table A1). As speculated by a rate of *rrs* gene divergence of 1–2% per 50 million years in bacterial endosymbionts (Moran et al. 1993; Ochman et al. 1999), the divergence between supergroup B and supergroup O probably started more than 120 million years ago. While the divergence date of *B. tabaci* complex was speculated to start about 50 million years ago, much more recent than that of supergroup B and O *Wolbachia* (Boykin et al. 2013). The double infection of *Wolbachia* supergroups B and O in the same population indicates horizontal transmission of *Wolbachia*. Horizontal transmission of *Wolbachia* has often been speculated based on phylogenetic analysis (Werren et al. 1995a; Sintupachee et al. 2006; Stahlhut et al. 2010; Schuler et al. 2013; Zhang et al. 2013). *Wolbachia* has also been reported from other whitefly genera such as *Trialeurodes* and some parasitoids (Raychoudhury et al. 2009; Cass et al. 2014), and this diversity of distribution may also hint horizontal transmission. Sintupachee et al. (2006) hypothesized that the horizontal transmission of *Wolbachia* from whiteflies to other arthropods may occur through plants, because whiteflies could feed on plants without ruining plant cells. Caspi-Fluger et al. (2011) presented a case study of horizontal transmission of *Rickttesia* in whiteflies via plants. Though we are yet unable to speculate on the origin of *Wolbachia* in whiteflies, we suggest that horizontal transmission of *Wolbachia* in whiteflies via plants warrants investigation especially as this bacterium has been detected outside of the bacteriocytes in the insect hosts.

## Conclusion

We conducted a comprehensive screening for *Wolbachia* in whiteflies, and the findings have broadened substantially the host spectrum of *Wolbachia* and revealed a new supergroup of *Wolbachia* in whiteflies. Our study also shows the limitations of protein-coding genes as molecular markers for *Wolbachia* investigation. Both specific and efficient molecular markers are needed for intensive surveys of *Wolbachia*. *Wolbachia* are transmitted vertically and horizontally in whiteflies. Clarifying the *Wolbachia* strains of whiteflies and their biological functions may provide novel clues for the development of efficient control technologies against invasive whiteflies and whitefly-transmitted plant viruses.

## Figures and Tables

**Figure A13:**
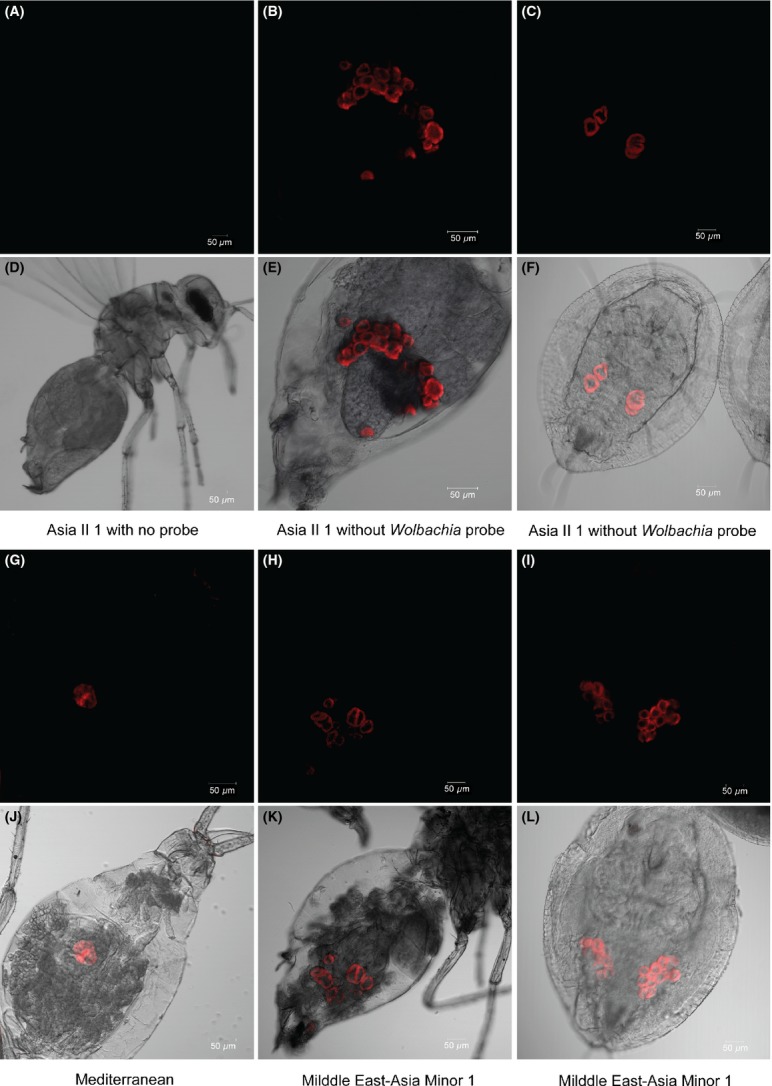
FISH controls. No probe control: Asia II 1; No *Wolbachia* probe control: Asia II 1; *Wolbachia*-free whiteflies control: Mediterranean (harboring *Portiera* and *Hamiltonella*) and Middle East-Asia Minor 1 (harboring *Portiera*, *Hamiltonella* and *Rickettsia*; Bing et al. 2013a). A–C, G–I: Overlay of channels of *Portiera* (red) and *Wolbachia* (green); D–F, J–L: Overlay of channels of *Portiera* (red), *Wolbachia* (green) and white light.

## Appendix

**Table A1:** Details of screen of *Bemisia afer* and *B. tabaci* cryptic species for *Wolbachia*. Rates of infections do not differ between sexes in each of the populations analyzed with Fisher's exact test (Populations with fewer than 10 individuals were excluded from the analysis)

							Sample size			Infection rate (%)^2^		
								
No.	Whitefly species	Location	Latitude	Longitude	Collection date	Host plant (family)^1^	F	M	UN	Females	Males	Overall
1	*Bemisia afer*	Linyi, Shandong, China	35°47′N	118°37′E	July 2012	*Broussonetia papyrifera* (1)	24	16	–	87.50	62.50	77.50
*Bemisia tabaci*												
2^3^	MED	Hefei, Anhui, China	31°95′N	117°48′E	October 2009	*Solanum melongena* (2)	21	8	–	9.52	0.00	6.90
3	MED	Hefei, Anhui, China	31°95′N	117°48′E	October 2009	*Solanum lycopersicum* (2)	17	13	–	0.00	0.00	0.00
4	MEAM1	Hefei, Anhui, China	31°92′N	117°14′E	October 2009	*Salvia splendens* (3)	21	7	–	0.00	0.00	0.00
5	MED	Nanjing, Jiangsu, China	32°02′N	118°54′E	September 2009	*Solanum melongena* (2)	25	5	–	0.00	0.00	0.00
6	MED	Nanjing, Jiangsu, China	32°02′N	118°54′E	September 2009	*Brassica oleracea var. capitata* (4)	15	15	–	0.00	0.00	0.00
7	MEAM1	Chongmingdao, Shanghai, China	31°50′N	121°80′E	November 2009	*Brassica oleracea var. capitata* (4)	16	11	–	0.00	0.00	0.00
8	MED	Chongmingdao, Shanghai, China	31°50′N	121°80′E	November 2009	*Capsicum annuum* (2)	21	10	–	0.00	0.00	0.00
9^4^	Asia II 3	Hangzhou, Zhejiang, China	30°23′N	120°18′E	April 2009	*Glycine max* (5)	26	21	–	0.00	0.00	0.00
10^3^,^4^	Asia II 1	Hangzhou, Zhejiang, China	29°27′N	119°18′E	October 2010	*Gossypium hirsutum* (6)	29	15	–	100.00	100.00	100.00
11^4^	China 1	Hangzhou, Zhejiang, China	30°23′N	120°18′E	November 2009	*Solanum lycopersicum* (2)	23	29	–	100.00	82.76	90.38
12^4^	MED	Ningbo, Zhejiang, China	29°48′N	121°35′E	June 2009	*Capsicum annuum* (2)	22	16	–	0.00	0.00	0.00
13	MED	Taizhou, Zhejiang, China	28°30′N	121°34′E	October 2012	*Cucurbita moschata* (7)	22	11	–	31.82	9.09	24.24
14^4^	MEAM1	Wenzhou, Zhejiang, China	27°47′N	120°39′E	September 2008	*Solanum melongena* (2)	20	20	–	0.00	0.00	0.00
15	MED	Nanchang, Jiangxi, China	28°72′N	115°91′E	October 2009	*Cucurbita moschata* (7)	16	11	–	0.00	0.00	0.00
16	MED	Nanchang, Jiangxi, China	28°72′N	115°91′E	October 2009	*Ipomoea batatas* (8)	16	14	–	0.00	0.00	0.00
17	MED	Nanchang, Jiangxi, China	28°72′N	115°91′E	October 2009	*Brassica campestris ssp. Pekinensis* (4)	21	8	–	0.00	0.00	0.00
18	MED	Nanchang, Jiangxi, China	28°72′N	115°91′E	October 2009	*Humulus scandens* (9)	16	12	–	0.00	0.00	0.00
19	MED	Nanchang, Jiangxi, China	28°29′N	116°01′E	October 2009	*Citrullus lanatus* (7)	13	16	–	0.00	0.00	0.00
20	MED	Nanchang, Jiangxi, China	28°29′N	116°01′E	October 2009	*Ipomoea batatas* (8)	20	7	–	0.00	0.00	0.00
21	MED	Jiujiang, Jiangxi, China	29°76′N	115°79′E	October 2009	*Capsicum annuum* (2)	16	11	–	0.00	0.00	0.00
22	MED	Jiujiang, Jiangxi, China	29°76′N	115°79′E	October 2009	*Ipomoea batatas* (8)	24	6	–	0.00	0.00	0.00
23	MED	Jiujiang, Jiangxi, China	29°76′N	115°79′E	October 2009	*Cucumis sativus* (7)	20	8	–	0.00	0.00	0.00
24	MED	Jiujiang, Jiangxi, China	29°76′N	115°79′E	October 2009	*Solanum melongena* (2)	13	2	–	0.00	0.00	0.00
25	MED	Jiujiang, Jiangxi, China	29°76′N	115°79′E	October 2009	*Phaseolus vulgaris* (5)	16	11	–	0.00	0.00	0.00
26^4^	Asia II 7	Guangzhou, Guangdong, China	23°09′N	113°21′E	October 2007	*Gossypium hirsutum* (6)	30	8	–	100.00	87.50	97.37
27^4^	Asia I	Zhaoqing, Guangdong, China	23°56′N	112°1′E	November 2010	*Ipomoea batatas* (8)	20	20	–	100.00	100.00	100.00
28^3^	Asia II 1	Zhaoqing, Guangdong, China	23°56′N	112°1′E	August 2012	*Arachis hypogaea* (5)	31	12	–	96.77	83.33	93.02
29^3^	Asia II 1	Zhaoqing, Guangdong, China	23°56′N	112°1′E	August 2012	*Ipomoea batatas* (8)	32	3	–	96.88	100.00	97.14
30^3^	Asia II 1	Sanya, Hainan, China	18°24′N	109°42′E	August 2009	*Ipomoea batatas* (8)	24	5	–	100.00	80.00	96.55
31	MEAM1	Nanning, Guangxi, China	22°38′N	108°23′E	August 2009	*Vigna unguiculata* (5)	17	13	–	0.00	0.00	0.00
32	MEAM1	Nanning, Guangxi, China	22°38′N	108°23′E	August 2009	*Gossypium hirsutum* (6)	22	0	–	0.00	–	0.00
33	MEAM1	Nanning, Guangxi, China	22°38′N	108°23′E	August 2009	*Cucumis sativus* (7)	17	13	–	0.00	0.00	0.00
34	MED	Beihai, Guangxi, China	21°29′N	109°09′E	August 2009	*Ipomoea batatas* (8)	10	12	–	0.00	0.00	0.00
35	Asia II 6	Baise, Guangxi, China	22°94′N	108°54′E	August 2009	*Luffa cylindrica* (7)	15	3	–	53.33	0.00	44.44
36	MEAM1	Baise, Guangxi, China	23°52′N	106°37′E	August 2009	*Vigna unguiculata* (5)	4	9	–	0.00	0.00	0.00
37	MEAM1	Baise, Guangxi, China	23°45′N	106°47′E	August 2009	*Benincasa hispida* (7)	15	12	–	0.00	0.00	0.00
38^4^	Asia II 6	Baise, Guangxi, China	22°94′N	108°54′E	November 2011	*Ipomoea batatas* (8)	36	6	–	13.89	16.67	14.29
39^4^	Asia I	Honghe, Yunnan, China	24°38′N	103°46′E	November 2011	*Ipomoea batatas* (8)	46	37	–	97.83	100.00	98.80
40	MED	Guiyang, Guizhou, China	26°40′N	106°67′E	July 2009	*Glechoma longituba* (3)	9	21	–	0.00	0.00	0.00
41	China 1	Zunyi, Guizhou, China	27°39′N	107°70′E	July 2009	*Solanum melongena* (2)	24	6	–	75.00	83.33	76.67
42	China 1	Zunyi, Guizhou, China	27°39′N	107°70′E	July 2009	*Ipomoea batatas* (8)	23	7	–	100.00	85.71	96.67
43^4^	Asia II 9	Shaoyang, Hunan, China	26°59′N	111°16′E	October 2011	*Ipomoea batatas* (8)	19	16	–	89.47	87.50	88.57
44	MED	Jishou, Hunan, China	28°18′N	109°38′E	September 2009	*Raphanus sativus* (4)	7	8	–	0.00	0.00	0.00
45	MED	Luoyang, Henan, China	34°46′N	112°46′E	September 2009	*Ipomoea batatas* (8)	16	5	–	0.00	0.00	0.00
46	MEAM1	Luoyang, Henan, China	34°66′N	112°51′E	September 2009	*Gossypium hirsutum* (6)	15	15	–	0.00	0.00	0.00
47	MEAM1	Luoyang, Henan, China	34°59′N	112°58′E	September 2009	*Solanum melongena* (2)	20	8	–	0.00	0.00	0.00
48	MEAM1	Luoyang, Henan, China	34°59′N	112°58′E	September 2009	*Cucumis sativus* (7)	26	4	–	0.00	0.00	0.00
49-1	Asia II 3	Zhengzhou, Henan, China	34°78′N	113°66′E	September 2009	*Solanum melongena* (2)	0	1	–	–	100.00	100.00
49-2	MED	Zhengzhou, Henan, China	34°78′N	113°66′E	September 2009	*Solanum melongena* (2)	18	9	–	0.00	0.00	0.00
50	MEAM1	Zhengzhou, Henan, China	34°78′N	113°66′E	September 2009	*Cucumis sativus* (7)	27	2	–	0.00	0.00	0.00
51	MED	Xinxiang, Henan, China	35°47′N	113°75′E	September 2009	*Phaseolus vulgaris* (5)	12	4	–	0.00	0.00	0.00
52	MED	Xinxiang, Henan, China	35°47′N	113°75′E	September 2009	*Raphanus sativus* (4)	26	3	–	0.00	0.00	0.00
53	MED	Xinxiang, Henan, China	35°47′N	113°75′E	September 2009	*Brassica campestris ssp. Pekinensis* (4)	20	8	–	0.00	0.00	0.00
54	Australia	Bundaberg, Queensland, Australia	24°48′S	152°27′E	–	*Euphorbia cyathophora* (10)	–	–	11	–	–	100.00

F, female adult; M, male adult; UN, unknown sex; –, not ascertained.

1 In all 22 species of host plants from 10 families, figures in parentheses indicate the names of the families: (1), Moraceae; (2), Solanaceae, (3), Lamiaceae, (4), Cruciferae, (5), Fabaceae, (6), Malvaceae, (7), Cucurbitaceae, (8), Convolvulaceae, (9), Cannabaceae, (10), Euphorbiaceae.

2 Infection rates of *Wolbachia* detected by diagnostic PCR of *rrs* gene.

3 Populations for the detection of *Wolbachia* supergroup O (Table 3).

4 Populations maintained in the laboratory on cotton since collection.

**Table A2:** List of the primers used for screening and sequencing

Gene	Hypothetical product	Primer name	Primer sequences (5′-3′)	Tm	Product size	Reference
*Bemisia* spp.						
*mtCOI*	Mitochondrial cytochrome oxidase subunit I	COI-F-C1-J-2195:	TTGATTTTTTGGTCATCCAGAAGT	54°C	759 bp	Frohlich et al. (1999)
		COI-R-TL2-N-3014:	TCCAATGCACTAATCTGCCATATTA			
Universal bacteria						
*rrs*	Ribosomal RNA 16S	27F:	AGAGTTTGATCMTGGCTCAG	50°C	1417 bp	Weisburg et al. (1991)
		1494R:	CTACGGCTACCTTGTTACGA			
*Wolbachia* spp.						
*rrs*	Ribosomal RNA 16S	Wol-16S-F:	CGGGGGAAAAATTTATTGCT	55°C	589 bp	Heddi et al. (1999)
		Wol-16S-R:	AGCTGTAATACAGAAAGTAAA			
*gatB*	Glutamyl-tRNA(Gln) amidotransferase, subunit B	gatB_F1:	GAKTTAAAYCGYGCAGGBGTT	54°C	471 bp	Baldo et al. (2006)
		gatB_R1:	TGGYAAYTCRGGYAAAGATGA			
*coxA*	Cytochrome coxidase, subunit I	coxA_F1:	TTGGRGCRATYAACTTTATAG	54°C	487 bp	Baldo et al. (2006)
		coxA_R1:	CTAAAGACTTTKACRCCAGT			
*hcpA*	Conserved hypothetical protein	hcpA_F1:	GAAATARCAGTTGCTGCAAA	54°C	515 bp	Baldo et al. (2006)
		hcpA_R1:	GAAAGTYRAGCAAGYTCTG			
*ftsZ*	Cell division protein	ftsZ_F1:	ATYATGGARCATATAAARGATAG	54°C	524 bp	Baldo et al. (2006)
		ftsZ_R1:	TCRAGYAATGGATTRGATAT			
*fbpA*	Fructose-bisphosphate aldolase	fbpA_F1:	GCTGCTCCRCTTGGYWTGAT	59°C	509 bp	Baldo et al. (2006)
		fbpA_R1:	CCRCCAGARAAAAYYACTATTC			
*wsp*	Outer surface protein	wsp_F1:	GTCCAATARSTGATGARGAAAC	59°C	546 bp	Baldo et al. (2006)
		wsp_R1:	CYGCACCAAYAGYRCTRTAAA			
*groEL*	Chaperonin GroEL	groEL-F:	CAACRGTRGSRRYAACTGCDGG	54°C	491 bp	Ros et al. (2009)
		groEL-R:	GATADCCRCGRTCAAAYTGC			
*gltA*	Citrate synthase	WgltAF1:	TACGATCCAGGGTTTGTTTCTAC	54°C	659 bp	Casiraghi et al. (2005)
		WgltARev2:	CATTTCATACCACTGGGC			

**Table A4:** Taxonomic details of *Wolbachia* hosts and the GenBank accession numbers of sequences included in the analysis

Phylum	Class	Order	Host species	16S rRNA gene	*gltA*	*groEL*	Supergroup
Arthropoda	Insecta	Hymenoptera	*Muscidifurax uniraptor*	L02882	–	–	A
Arthropoda	Insecta	Hymenoptera	*Nasonia vitripennis*	M84688	AY714795	AY714812	A
Arthropoda	Prostigmata	Acarina	*Bryobia sarothamni*	EU499315	–	EU499330	B
Arthropoda	Prostigmata	Acarina	*Bryobia praetiosa*	EU499317	EU499327	EU499332	B
Arthropoda	Insecta	Hymenoptera	*Nasonia vitripennis*	M84686	AY714782	AY714796	B
Arthropoda	Insecta	Hemiptera	*Bemisia tabaci*	JN204507	–	–	B
Nematoda	Secernentea	Spirurida	*Onchocerca ochengi*	AJ010276	AJ609640	–	C
Nematoda	Secernentea	Spirurida	*Onchocerca gibsoni*	AJ276499	AJ609639	AJ609652	C
Nematoda	Secernentea	Spirurida	*Dirofilaria repens*	AJ276500	–	AJ609653	C
Nematoda	Secernentea	Spirurida	*Dirofilaria immitis*	Z49261	AJ609641	–	C
Nematoda	Chromadorea	Spirurida	*Brugia malayi*	AF051145	AJ609643	AE017321	D
Nematoda	Secernentea	Spirurida	*Litomosoides sigmodontis*	AF069068	AJ609645	AF409113	D
Arthropoda	Collembola	Collembola	*Folsomia candida*	AF179630	AJ609649	–	E
Arthropoda	Ellipura	Collembola	*Mesaphorura macrocheta*	AJ422184	–	–	E
Arthropoda	Insecta	Neuroptera	*Myrmeleon mobilis*	DQ068882	–	–	F
Arthropoda	Insecta	Isoptera	*Kalotermes flavicollis*	Y11377	AJ609651	AJ609660	F
Nematoda	Secernentea	Spirurida	*Mansonella ozzardi*	AJ279034	–	AJ609657	F
Arthropoda	Insecta	Isoptera	*Zootermopsis nevadensis*	AY764280	AY764282	AY764277	H
Arthropoda	Insecta	Siphonaptera	*Ctenocephalides felis*	AY335923	AJ609650	AJ609659	I
Arthropoda	Insecta	Siphonaptera	*Orchopeas leucopus*	AY335924	–	–	I
Nematoda	Secernentea	Spirurida	*Dipetalonema gracile*	AJ548802	AJ609648	AJ609658	J
Arthropoda	Arachnida	Prostigmata	*Bryobia* sp.	EU499316	EU499326	EU499331	K
Nematoda	Phasmida	Tylenchida	*Radopholus similis*	EU833482	–	EU833484	L
Arthropoda	Insecta	Hemiptera	*Tuberolachnus salignu*	JN384085	–	–	M
Arthropoda	Insecta	Hemiptera	*Aphis* sp.	JN384091			M
Arthropoda	Insecta	Hemiptera	*Cinara cedri*	–	–	JN384053	M
Arthropoda	Insecta	Hemiptera	*Toxoptera aurantii*	JN384094	–	–	N
Arthropoda	Insecta	Hemiptera	*Toxoptera aurantii*	JN384095	–	–	N
**Arthropoda**	**Insecta**	**Hemiptera**	***Bemisia tabaci***	**KF454771**	**KF587270**	**KF452543**	**O**

**Table A6:** Divergence between the *Wolbachia* detected in the putative species Asia II 1 of the *B. tabaci* complex (*wBt*_10) and other supergroups based on 16S rRNA gene sequences

Host species	Supergroup	Divergence (%)	GenBank accession no.
*Muscidifurax uniraptor*	A	3.73	L02882
*Nasonia vitripennis*	A	3.64	M84688
*Bryobia sarothamni*	B	4.98	EU499315
*Bryobia praetiosa*	B	4.98	EU499317
*Bemisia tabaci*	B	5.18	JN204507
*Nasonia vitripennis*	B	4.83	M84686
*Onchocerca ochengi*	C	5.61	AJ010276
*Onchocerca gibsoni*	C	5.44	AJ276499
*Dirofilaria repens*	C	5.99	AJ276500
*Dirofilaria immitis*	C	5.63	Z49261
*Brugia malayi*	D	4.59	AF051145
*Litomosoides sigmodontis*	D	5.15	AF069068
*Folsomia candida*	E	3.65	AF179630
*Mesaphorura macrocheta*	E	4.04	AJ422184
*Mansonella ozzardi*	F	4.95	AJ279034
*Myrmeleon mobilis*	F	3.90	DQ068882
*Kalotermes flavicollis*	F	3.70	Y11377
*Zootermopsis nevadensis*	H	4.41	AY764280
*Ctenocephalides felis*	I	7.22	AY335923
*Orchopeas leucopus*	I	7.48	AY335924
*Dipetalonema gracile*	J	5.59	AJ548802
*Bryobia sp*.	K	3.25	EU499316
*Radopholus similis*.	L	4.79	EU833482
*Tuberolachnus salignu*	M	2.52	JN384085
*Aphis sp*.	M	2.59	JN384091
*Toxoptera aurantii*	N	4.50	JN384094
*Toxoptera aurantii*	N	4.80	JN384095
